# Estimating the impact of divergent mating phenology between residents and migrants on the potential for gene flow

**DOI:** 10.1002/ece3.5001

**Published:** 2019-03-12

**Authors:** Colin Bonner, Nina A. Sokolov, Sally Erin Westover, Michelle Ho, Arthur E. Weis

**Affiliations:** ^1^ Department of Ecology and Evolutionary Biology University of Toronto Toronto ON Canada; ^2^ Koffler Scientific Reserve at Jokers Hill University of Toronto Toronto ON Canada

**Keywords:** assisted gene flow, assortative mating, evolutionary rescue, flowering time, hybridization, temporal reproductive isolation

## Abstract

Gene flow between populations can allow the spread of beneficial alleles and genetic diversity between populations, with importance to conservation, invasion biology, and agriculture. Levels of gene flow between populations vary not only with distance, but also with divergence in reproductive phenology. Since phenology is often locally adapted, arriving migrants may be reproductively out of synch with residents, which can depress realized gene flow. In flowering plants, the potential impact of phenological divergence on hybridization between populations can be predicted from overlap in flowering schedules—the daily count of flowers capable of pollen exchange—between a resident and migrant population. The accuracy of this prospective hybridization estimate, based on parental phenotypes, rests upon the assumptions of unbiased pollen transfer between resident and migrant active flowers. We tested the impact of phenological divergence on resident–migrant mating frequencies in experiments that mimicked a single large gene flow event. We first prospectively estimated mating frequencies two lines of *Brassica rapa*selected or early and late flowering. We then estimated realized mating frequencies retrospectively through progeny testing. The two estimates strongly agreed in a greenhouse experiment, where procedures ensured saturating, unbiased pollination. Under natural pollination in the field, the rate of resident–migrant mating, was lower than estimated by phenological divergence alone, although prospective and retrospective estimates were correlated. In both experiments, differences between residents and migrants in flowering schedule shape led to asymmetric hybridization. Results suggest that a prospective estimate of hybridization based on mating schedules can be a useful, although imperfect, tool for evaluating potential gene flow. They also illustrate the impact of mating phenology on the magnitude and symmetry of reproductive isolation.

## 1 INTRODUCTION

Gene flow between populations has important consequences for the evolution, survival, and spread of species. It can introduce adaptive alleles into a declining resident populations, potentially saving it from extinction through genetic rescue (Frankham, 2015; Tallmon, Luikart, & Waples, 2004). One conservation measure—Assisted Gene Flow (AGF), has been proposed to affect genetic rescue of populations threatened by global change by introducing adaptive alleles from related population in situ (Aitken & Whitlock, 2013). On the other hand, a small locally adapted population with distinct genetic combinations can be “swamped out” by the inflow of genetic material from a larger population (Ellestrand, Prentice, & Hancock, 1999; Lenormand, 2002). Finally, gene flow into a species invading a new community can increase the species' invasiveness by introducing alleles that increase their survival, reproductive rate, or dispersal ability in their newly invaded environment (Ellestrand et al., 1999, Ellstrand & Schierenbeck 2000). Crucially, consequences of gene flow dependent not only on migration rates, but also on successful interbreeding between residents and migrants. Successful interbreeding, in turn, depends on synchrony of resident and migrant reproductive phenologies.

Many species show geographic variation in reproductive phenology (e.g., Conklin, Battley, Potter, & Fox, 2010; Dambroski & Feder, 2007; Eckhart, Geber, & McGuire, 2004; Guttman, Bramble, & Sexton, 1991, Hall & Willis, 2006) These populations are thus not only isolated by distance, but also by time (Wright, 1943). In seasonally breeding species, even adjacent populations can be in reproductive isolation if their mating periods are asynchronous. For instance, flowering periods do not overlap between cultivated sunflowers and the weedy sunflowers growing in the vicinity, impeding gene flow between the two (Burke, Gardner, & Rieseberg, 2002).The apparent widespread nature of phenological divergence makes it likely to impact the dynamics of invasiveness, the efficacy of conservation practices, and the evolution of new species by reducing the potential of populations to interbreed.

The potential for divergent mating phenology to bias and impede gene flow in plants has been recently investigated (Barbour, Potts, Vaillancourt, & Tibbits, 2006; Wadgymar & Weis, 2017; Weis, 2015). Two plants can exchange pollen only if they are in flower at the same time. Most plants produce multiple flowers over a period of days, and so the mating probability between any two individuals can be estimated by the overlap in their flowering schedules. Wadgymar and Weis (2017) explored this by mimicking the first stage of an AGF program. Wadgymar and Weis grew northern “resident” Minnesota population of the prairie annual *Chamecrista fasiculata* alongside “migrants” from several potential source populations and estimated the potential for pollen exchange form their flowering schedules. The southern‐most migrant population, sourced from North Carolina, came into flower ~24 days behind the residents when grown at the northern latitude. By the time the migrants reached peak flower production, the residents had already advanced to fruit maturation. Even though the residents and migrants were planted in equal numbers, fewer than 3% of the resident flowers could have received North Carolina migrant pollen, and reciprocally, less than 1% of the migrant flowers could have received resident pollen. Repeating the same procedure with a migrant population sourced from Pennsylvania, which flowered ~10 days after the residents, they found higher expected hybridization rates of 31% and 38% for residents and migrants, respectively, but these were still well short of the 50% expected under phenological matching.

The flowering schedule method devised by Weis and Wadgymar (2017) is simple to employ when designing AGF programs, but its predictive value rests on the assumption that on each day of the flowering season, every open flower has an equal probability of exchanging pollen with every other open flower. If pollinators transfer pollen between residents and migrants non‐randomly, realized hybridization can differ from that predicted by phenological overlap alone. Gametic incompatibilities between residents and migrants could further cause realized hybridization rates to diverge from phenological expectations. The degree of bias can be evaluated by comparing the resident–migrant hybridization rate estimated prospectively form flowering schedules to a retrospective estimate based on progeny testing. Quite simply, with unbiased pollination (and complete gametic compatibility) resident plants should produce resident–migrant offspring it proportion to the number of contemporaneous mating opportunities they shared with migrants.

We performed a pair of two‐generation experiments to mimic the translocation of phenologically mismatched migrants into a resident population. These experiments were designed to mimic an AGF program, whereby a single massive gene flow event is imposed upon a resident population in need of genetic rescue. In both experiments, the potential for hybridization was estimated in the first generation from overlap in flowering schedules of the phenologically divergent lines—the prospective estimate. The realized hybridization rate was revealed in the progeny generation through quantitative genetic methods. Specifically, the genotypes of the progeny generation were expected to include purebred residents, purebred migrants, and F_1_ hybrids between the two. The proportion of successful hybrid matings by residents was determined retrospectively by comparing the flowering time distribution of their progeny to the distributions of known residents and hybrids, as detailed below. We found that realized hybridization rates were accurately predicted form phenological overlap in a greenhouse experiment, where procedures ensured random and saturating pollen loads to all flowers. Although prospective and retrospective estimates were correlated under natural pollination in the field, the rate of resident–migrant mating was lower than predicted by phenological divergence alone.

## 2 MATERIAL AND METHODS

### 2.1 Resident and migrant strains

We used two lines of *Brassica rapa* selected for divergent bolting time (Austen & Weis, 2015) as a model system for phenological mismatch between residents and migrants in an AGF program. This species is a self‐incompatible annual. The founding stock for the lines was collected from a large natural Quebec population (see Austen & Weis, 2015). After two generations of selection the flowering times of the two lines differed by about two weeks under field conditions, with the earlier line flowering ~30 d after sowing and the later ~43 d. We will refer to the earlier flowering line as the “resident” population in need of evolutionary rescue through AGF, with the “migrant” population flowering later.

### 2.2 Experiment 1: Hybridization under controlled pollination

The first experiment was designed to test if prospective estimates of hybridization, derived solely from flowering schedules, agreed with retrospective estimates, derived through progeny testing. It was performed in the greenhouse, and employed daily, random, hand pollen transfer. These are the conditions most likely to produce agreement between the two estimates. Failure to find agreement in this experiment would conclusively show that the Wadgymar and Weis (2017) method is invalid, and factors other than simply the number of open flowers are important in determining siring rates in this system.

We grew 25 focal plants of each phenological line in the University of Toronto greenhouse in spring 2015. Seeds were sown into 8 × 8 × 34 cm pots, filled with a 70:30 mixture of potting soil and coarse sand. They were fertilized with 20:20:20 NPK fertilizer bi‐weekly. Each plant was inspected daily, and its date of first flower recorded. The number of open flowers on each focal plant was recorded twice weekly to construct the flowering schedules needed for the prospective estimate of hybridization.

**Figure 1:**
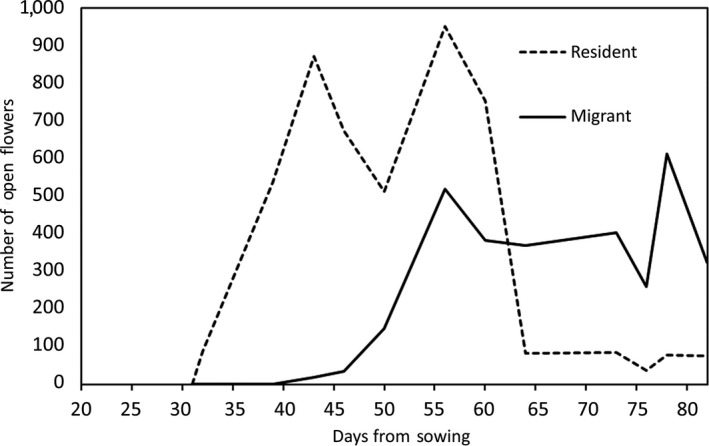
The flowering schedule data collected from sires in the ideal scenario, showing the number of flowers which were open on each sampling day as a number of days from planting

To assure random pollen exchange within each day, the focal plants were pollinated by taking two walks through the greenhouse every day, brushing all stigmas and anthers with a single feather. The second path retraced the first going to opposite direction, so as to minimize bias due to distance between plants. All plants should thus experience similar pollination environments and variations in pollen load and quality. The offspring of these plants were expected to be a mixture of hybrid F_1_s and purebreds, with the proportion of hybrids depending on the flowering schedule overlap between the populations (Figure 1).

**Figure 2:**
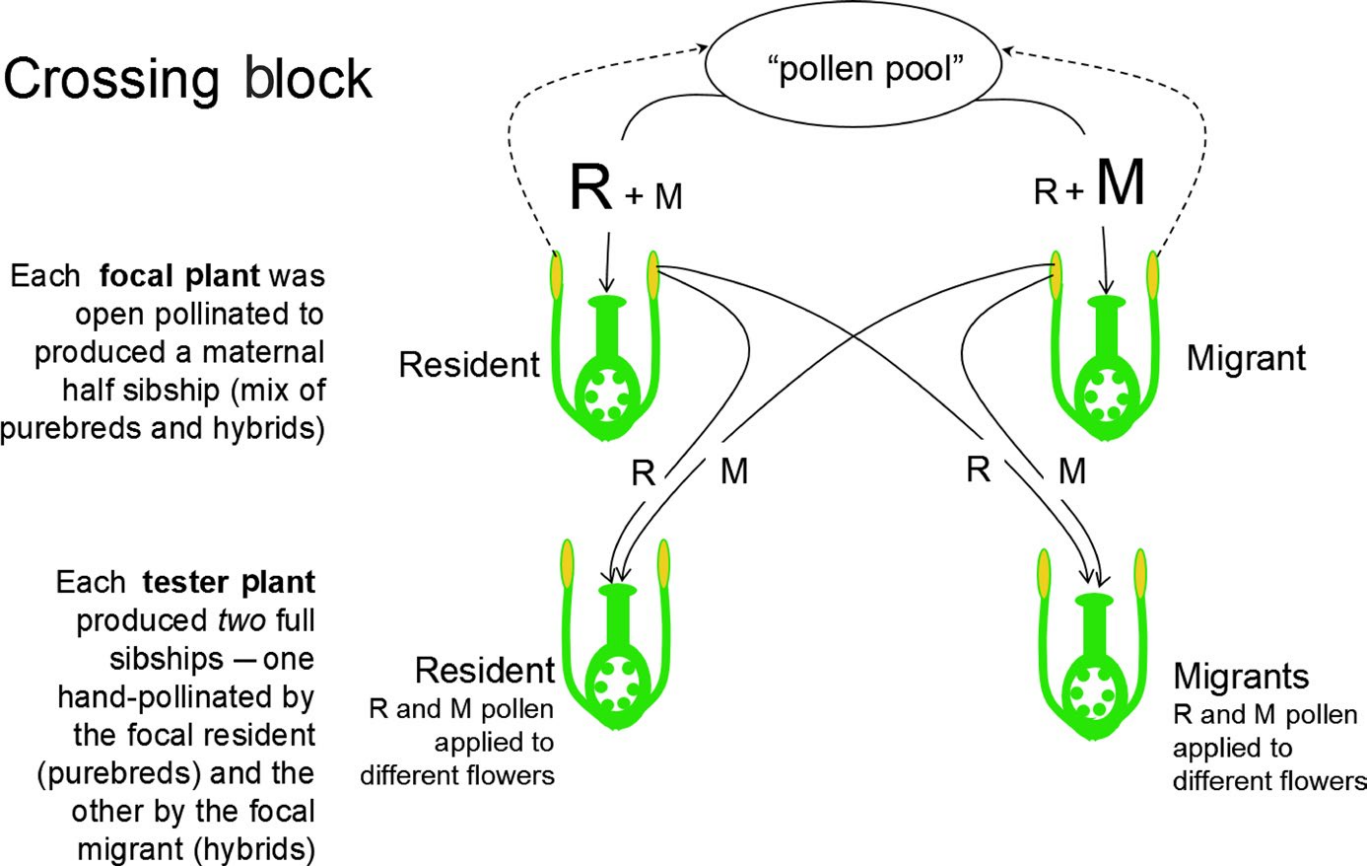
Crossing scheme of plants in experiment one: generation one. Each open‐pollinated plant produced a half sibship consisting of an unknown mixture of purebreds and hybrids sired by the “pollen cloud”, which itself was contributed to by all open‐pollinated plants in the greenhouse. Each controlled cross sired two full sibships, one hybrid and one purebred, sired by the open‐pollinated plants within the crossing block

The retrospective estimate of hybridization rate was made by comparing the flowering times of the open‐pollinated progeny to those of known purebreds and hybrids. These “known” plants were produced by assigning each focal plant to one of 25 crossing blocks (Figure 2), with one resident and one migrant in each. Each crossing block also included two additional “tester” plants, one a resident and the other a migrant. Plants were widely spaced to ensure no accidental contact between receptive flowers and no pollen vectors were observed in the greenhouse space, so that offspring can be attributed to designated pollen donors with high confidence. Several flowers on each tester plant were then hand pollinated with anthers taken from the two focal plants in the block. Thus, each tester plant produced one full sibship of offspring that were sired by a resident and another full sibship sired by a migrant (Figure 2). This gave four genotypic classes of offspring: purebred residents, purebred migrants, and the reciprocal F_1_s.

The phenotypic distributions for flowering time were then obtained for both (a) the open‐pollinated progeny seed produced on the focal plants and (b) the purebred and hybrid progeny sired by the focal plants in the controlled crosses. To obtain these distributions, we planted 55 open‐pollinated progeny from each focal plant, and 12 progeny from each successful hand‐cross. A total of 3,715 progeny were grown in October 2015, using 164‐ml “conetainer” pots (Stewe & Sons, Corvallis, OR) in the same greenhouse, and in the same soil mix as their parents. The date of first flowering was recorded for each plant. Details of the statistical analyses are given below.

### 2.3 Experiment 2: Hybridization under natural pollination

The second experiment, performed in the field, tested if flowering schedules adequately predict hybridization under natural pollination. Deviance of realized from potential hybrid matings could arise in several ways. For instance, pollinator preference for large inflorescences could cause plants at peak flower to contribute disproportionately to the successful pollen pool, unless there is a compensatory decline in the per‐flower visitation rate with display size (e.g., Brys & Jacquemyn, 2010; Dudash, Hassler, Stevens, & Fenster, 2011; Karron & Mitchell, 2011). At low plant density, pollinators typically move between adjacent plants (Fenster, 1991; Thomson & Thomson, 1989), but at high plant density, skip over near neighbors (Cresswell, 1997). Finally, flowers produced late in the flowering schedule are less likely to set seed (Austen, Forrest, & Weis, 2015; Ison & Wagenius, 2014), so that pollen donated late in the season is less likely to sire offspring. Thus, on any one day, the pool of successful gametes may be a biased subset of the overall gamete pool, decaying the relationship between potential and realized hybridization.

This experiment also tested the notion that hybridization declines as phenological mismatch increases. We manipulated mismatch between populations by staggering the planting date for the migrant line, which altered the calendar date of migrant first flowering, while maintaining the same date for residents.

In the summer of 2015, parental plants from the resident and migrant lines were exposed to natural pollinators at the Koffler Scientific Reserve at Joker's Hill (KSR), 58 km north of the University of Toronto. As of the summer of 2015, no wild population of *Brassica rapa*has been observed on the property. We applied three levels of mismatch. For the “Asynchronous Flowering” (mismatched) treatment, the two strains were planted on the same day (May 19th), so that the resident line would start to flower two weeks ahead of the migrant, as they naturally would. In the “Synchronous Flowering” treatment, migrants were planted 2 weeks ahead (May 6th) of the residents so that the two would come into flower on the same calendar date. For the “Intermediate” treatment, the migrant strain was planted only one week ahead of the resident. In addition to the three synchrony treatments, there were two control treatments, resident only and migrant only (expected hybridization = 0) planted on May 19th. These five treatments were replicated three times, for a total of 15 plots.

The experimental plants were started from seed in conetainer pots in the KSR greenhouse, using the same soil as above. The seeds were derived from the purebred controlled crosses performed as part of Experiment 1. Once all plants were established across all treatments, they were moved, in the containers, and sunk into 1.3 × 1.3 m experimental plots filled with local sand (see Austen & Weis, 2015) on May 23rd. Each plot in each treatment held 21 plants per line, randomly dispersed in a hexagonal grid, with one plant from each purebred full‐sibling family, so that experimental plots had a total of 42 plants and control plots had 21 plants. Thus, each plant in a plot had a full sibling in every other plot but no siblings in its own plot. Plots were separated by at least 200 meters to prevent pollen exchange between them, as in previous pollination experiments (Kunin, 1993; Mustajärvi, Siikamäki, Rytkönen, & Lammi, 2001).

Flowering began on June 13th and ended August 7th. Plants were censused every day during this interval, recording date of first flowering and the number of open flowers on each plant every day after. To determine if the proportion of flowers setting seed declines with plant age (see Austen & Weis, 2014) we tagged a subsample of flowers every 2–3 days and harvested seeds as they matured throughout August, stored them by maternal group, and counted the number of seeds from flowers that had been tagged.

Hybridization rate was estimated retrospectively from the flowering times of the offspring of the field‐grown, open‐pollinated plants. In the summer of 2016, we planted 11 seed offspring from each field plant on the roof of the Earth Sciences Center building at the University of Toronto, totaling 5,544 plants. Additionally, 48 families from the controlled crosses in experiment 1 were planted: 12 resident and 12 migrant purebred sibships, and F_1_ hybrid sibships from 12 resident and 12 migrant mothers, with 12 plants per sibship, for a total of 576 plants of known parentage. We used the same conetainer pots and soil as for the field generation.

### 2.4 Prospective estimate of hybridization

Our notation for the several hybridization estimates are as follows: *h_iR_*is the proportion of flowers on individual *i* of the resident population expected to be pollinated by migrants; *H_R_* is the mean of *h_iR_*, and constitutes the prospective estimate of the frequency of hybrids among the seed progeny produced by the resident population; *H'_R_*is the retrospective estimate for the frequency of hybrids among the seed progeny of residents, derived from progeny testing. The corresponding hybridization rates for migrant seed parents use the subscript *M*.

We calculated a prospective estimate of hybridization rate for each plant (Wadgymar & Weis 2017; Weis, 2015) using the following:

(1) $$h_{\mathrm{iR}}=\sum_{d=1}^{D}f_{\mathrm{iRd}}q_{d}$$

and

(2) $$h_{\mathrm{iM}}=\sum_{d=1}^{D}f_{\mathrm{iMd}}\left(1-q_{d}\right)$$

where *f_iRd_* and *f_iMd_* are the proportion of all flowers across the season that were counted on day *d*for resident or migrant *i*, respectively. The term *q_d_* is the proportion of pollen in the pollen pool on day *d* contributed by migrants, as proxied by the proportion of open flowers; this assumes that on each day, each stigma received a random sample of the pollen produced on that day, and that each flower contributed an equal amount of pollen. Again, the plot means of *h_iR_* and *h_iM_,* denoted as *H_R_* and *H_M_*, respectively, were our prospective estimates of hybridization.

### 2.5 Retrospective estimate of hybridization

Our strategy to retrospectively estimate hybridization rates, *H'_R_*and *H'_M_*, used flowering time itself as the marker for paternity. The two parental populations differed genetically for the number of days from emergence to flowering, with the F_1_ hybrids having intermediate flowering times (see data below). Seed offspring produced by resident plants in the open‐pollinated generation of each experiment will be a mixture of those sired by migrants and those sired by other residents. Thus, the phenotypic distribution of flowering times among all offspring of all resident mothers should be a composite, with proportion *H'_R_*being drawn from the flowering time distribution for hybrids, and 1‐* hr'_R_*drawn from the purebred distribution. Hybridization rate could thus be estimated by comparing the observed mixed distribution to a series of synthetic distributions constructed from the flowering time distributions of known purebred and hybrid progeny.

**Figure 3:**
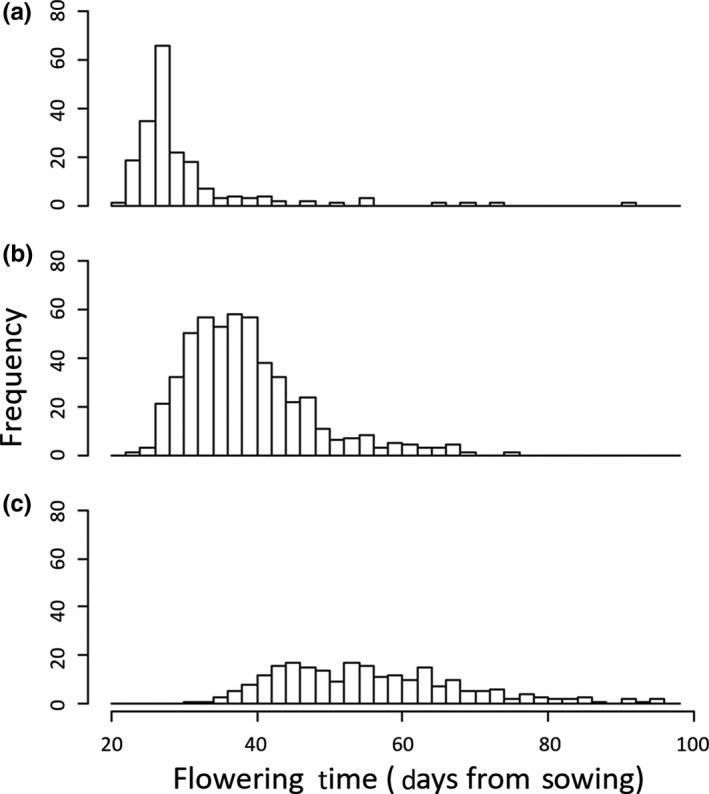
Histograms for flowering time of known crosses grown in the greenhouse, with purebred resident (a), F_1_ hybrids (b), and purebred migrants (c)

Specifically, the flowering time frequency distributions of the known hybrids and purebreds from Experiment 1 (Figure 3) were multiplied by a series of hypothetical hybridization rates, H_R_* and 1‐ H_R_*, respectively, then added together into a single synthetic distribution. H_R_*_,_the hypothetical hybridization rate, was varied from 0.5 (expected under phenological matching) down to 0.0 (complete mismatch). The observed distribution of the open‐pollinated offspring was then tested against each of the synthetic distributions with a χ^2^ goodness‐of‐fit test. The H_R_* for the synthetic distribution yielding the minimum χ^2^was interpreted as the retrospective estimate of hybridization rate, *H'_R_*, by resident plants through female function (See Appendix S1A). The same procedure was applied for seed offspring of migrant plants. Confidence intervals were generated by taking the H_j_* values corresponding to a 3.84 increase (the critical χ^2^ at one degree of freedom and *p* = 0.05) in the χ^2^value on either side of the minimum value.

A second method was also tried for retrospectively estimating *H'_R_*and *H'_M_* for the field experiment. A Linear Discriminant Analysis (hereafter LDA) was constructed using the known purebred and hybrid offspring from the controlled crosses. This LDA serves to test if the first method is corroborated by one already established in the literature. Separate functions were constructed to distinguish hybrids from purebred residents and from purebred migrants (see Appendix S1B). The functions were then applied to the open‐pollinated offspring to obtain the proportion classified as hybrids. In addition to flowering time, the LDA included stem height and diameter, number of stem nodes, and corolla width as marker traits. These traits correlate with flowering date, and so also differed between the resident and migrant lines. To obtain confidence intervals on these retrospective estimates, we ran 1,000 bootstrap samples from the open‐pollinated offspring through the LDA classification function and recorded the 2.5th and 97.5th percentile values to obtain confidence intervals. Known hybrids and purebreds were run through the LDA as a “training set” to determine how accurately it assigned genotype. The LDA was run using the MASS package in R (Venables & Ripley, 2002). To determine the relationship of fruiting success to plant age (days since start‐of‐flowering) we applied a poisson regression in R (R Development Core Team, 2016)

## 3 RESULTS

### 3.1 Hybridization under controlled pollination

In the parental generation of the controlled pollination experiment, resident plants came into flower an average of 28.86 days after sowing and the migrants 52.04 days. This 23‐day displacement in flowering schedules resulted in phenological mismatch between the two populations (Figure 1). The prospective estimates of hybridization were asymmetrical, with the resident *H_R_* being 0.216 and the migrant *H_M_* being 0.345. This asymmetry was due to differences in the shape of flowering schedules, as discussed below.

In the controlled crosses (used to construct the synthetic distributions for the retrospective estimate), the F_1_ hybrid flowering time was intermediate to the purebred lines (Figure 3). Mean flowering time of hybrids was 39.14 days after sowing, which was closer to residents (30.20) than to migrants (56.35) by 8.26 days, indicating modest directional dominance. Migrants had a higher variance in start‐of‐flowering, with the resident, hybrid, and migrant flowering time distributions having standard deviations of 8.84, 8.42, and 12.92, respectively.

**Figure 4:**
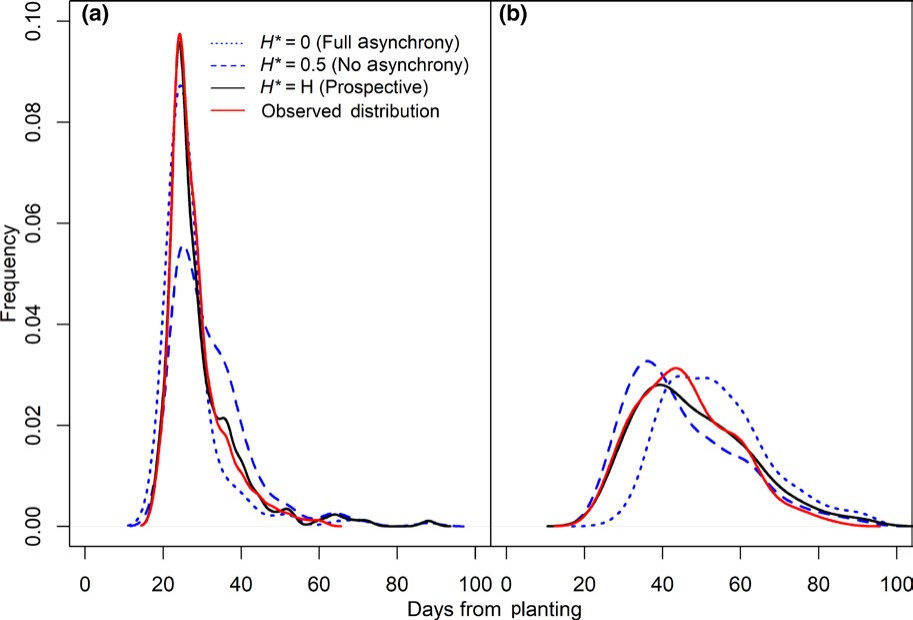
Example synthetic mixture distributions of purebred and hybrid flowering times for resident (a) and migrant (b) flowering lines in the greenhouse experiment. Plotted are the zero hybridization (H* = 0) and 50% hybridization (H* = 0.5) mixtures, the mixture predicted from the prospective estimate (H), and the mixture which best fits the progeny flowering time distribution (Observed Distribution)

**Figure 5:**
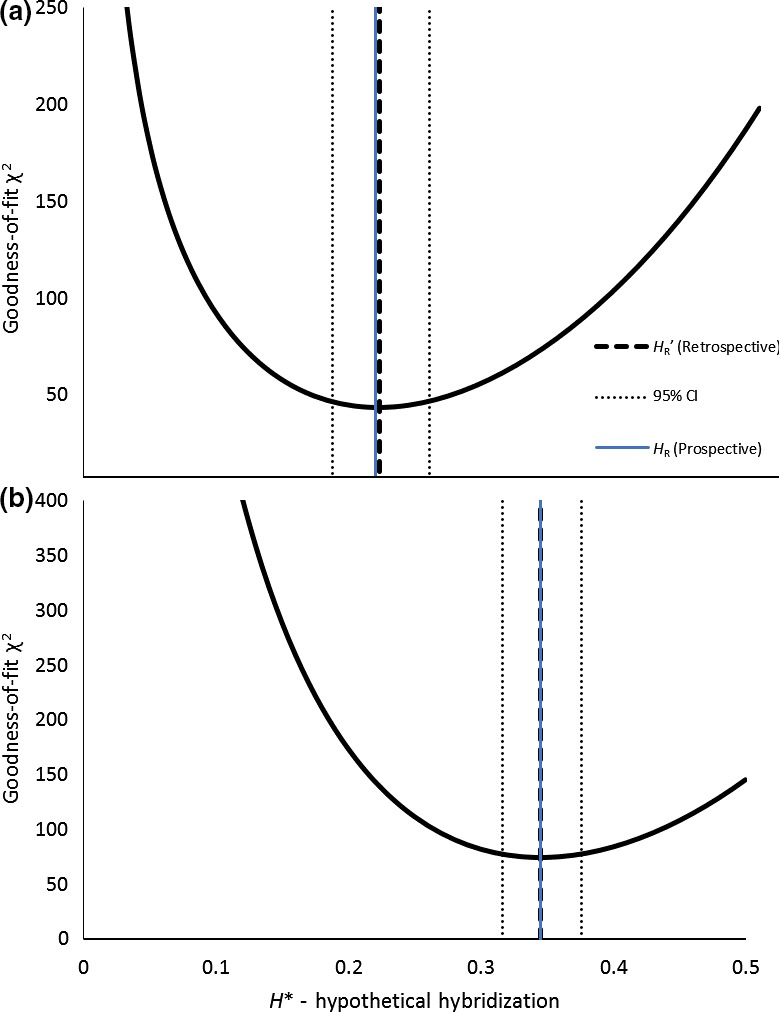
The χ^2^ distribution for early‐flowering resident (a) and late‐flowering migrant (b) plants, with the hybridization value at the minimum χ^2^ plotted

The retrospective estimates of hybridization strongly agreed with the prospective. Figure 4 compares the observed distributions of progeny flowering times to the distributions expected from the prospective hybridization estimates. For contrast, we also illustrate the distributions expected under phenological matching and under complete mismatch (H* = 0.5, and 0.0, respectively). The synthetic distributions giving the best fit to the observed were at *H'_R_* = 0.220 for resident progeny, and *H'_M_* = 0.346 for migrant (Figure 5). The strong agreement of the prospective estimate to the retrospective (0.216 vs. 0.220, and 0.345 vs. 0.346, for residents and migrants, respectively) indicates that the overlap in flowering schedules is the main determinant of hybridization rate when pollination is saturating, consistent, and random within days.

### 3.2 Hybridization under field conditions

Under natural pollination, and field growing conditions, there was weaker agreement between prospective and retrospective hybridization estimates. This section presents summary analyses of the entire experiment, focusing on 2 of the 15 experimental plots to illustrate several points.

**Figure 6:**
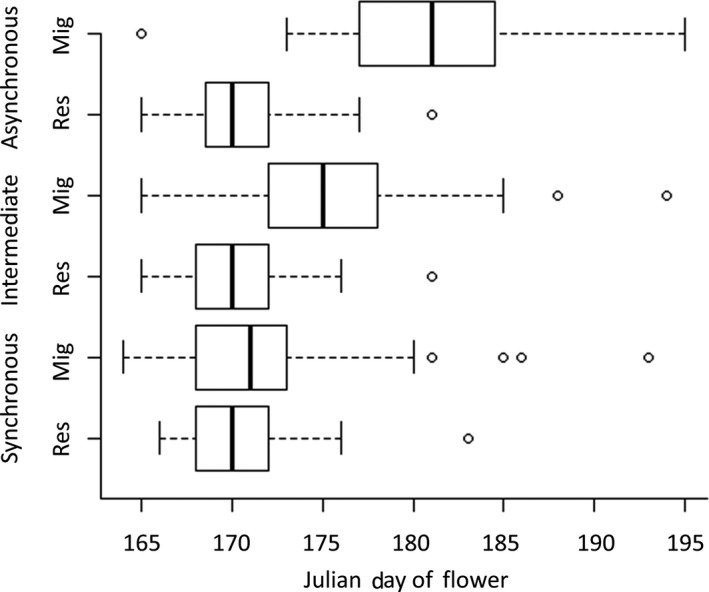
Boxplots showing the spread of start‐of‐flowering across treatments for both resident (Res) and migrant (Mig) lines

**Figure 7:**
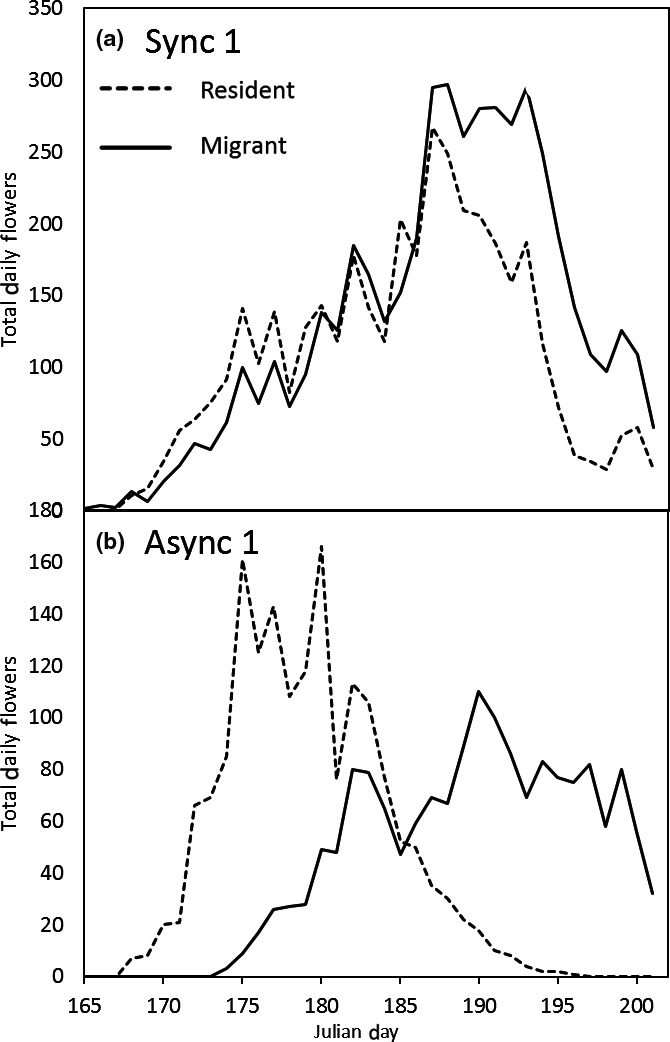
Flowering schedule for plots S1 (synchronous) and A1 (asynchronous) exemplifying the difference between treatments

Staggered sowing dates successfully altered the date of first flowering in the treatments (Figure 6), which in turn altered the overlap in resident and migrant flowering schedules. Figure 7 illustrates the flowering schedules for the most synchronous and the most asynchronous plots. In the former, both populations contribute more or less equally to the mating pool across the season, whereas in the later the resident population contributed predominantly early in the season and the migrant at the end. In the field, migrants produced more flowers than residents (Figure 7).

Under complete flowering synchrony, equal population size and equal flower production it is expected that *H_R_* = *H_M_* = 0.50, that is, mating is random between populations. For resident plants, the mean *H_R_* values were 0.520 ± 0.014, 0.456 ± 0.026, and 0.348 ± 0.025 in the synchronous, intermediate and asynchronous treatments, respectively (see Table 1). Thus, opportunity for hybridization by residents under asynchrony was ~68% of that under synchrony. Hybridization opportunity for migrant mothers was lower overall, but also declined with increasing mismatch. Mean values for *H_M_* were 0.388 ± 0.014, 0.312 ± 0.019, and 0.283 ± 0.027 for the three treatments (Table 1); opportunity for hybridization by migrants under asynchrony was 27% less than under synchrony. The reductions in migrant hybridization opportunity can be attributed to their greater late‐season flower production, which increased the potential for migrant‐migrant pollen exchange.

**Table 1:** Prospective estimates of hybridization rates for each plot across each treatment with treatment means

Treatment	Plot	Resident		Migrant	
		Mean	Grasnd mean	Mean	Grand mean
Synchronous	S1	0.518 ± 0.01		0.436 ± 0.010	
	S2	0.522 ± 0.015	0.520 ± 0.014	0.351 ± 0.016	0.388 ± 0.014
	S3	0.522 ± 0.016		0.378 ± 0.014	
Intermediate	I1	0.446 ± 0.032		0.293 ± 0.023	
	I2	0.413 ± 0.025	0.456 ± 0.026	0.298 ± 0.020	0.312 ± 0.019
	I3	0.509 ± 0.018		0.347 ± 0.012	
Asynchronous	A1	0.237 ± 0.019		0.267 ± 0.037	
	A2	0.349 ± 0.030	0.348 ± 0.025	0.282 ± 0.023	0.283 ± 0.027
	A3	0.459 ± 0.024		0.301 ± 0.016	

Note

Treatment had a significant effect (*p*«0.001, linear model in *R*), while plot and phenology did not**.**

**Figure 8:**
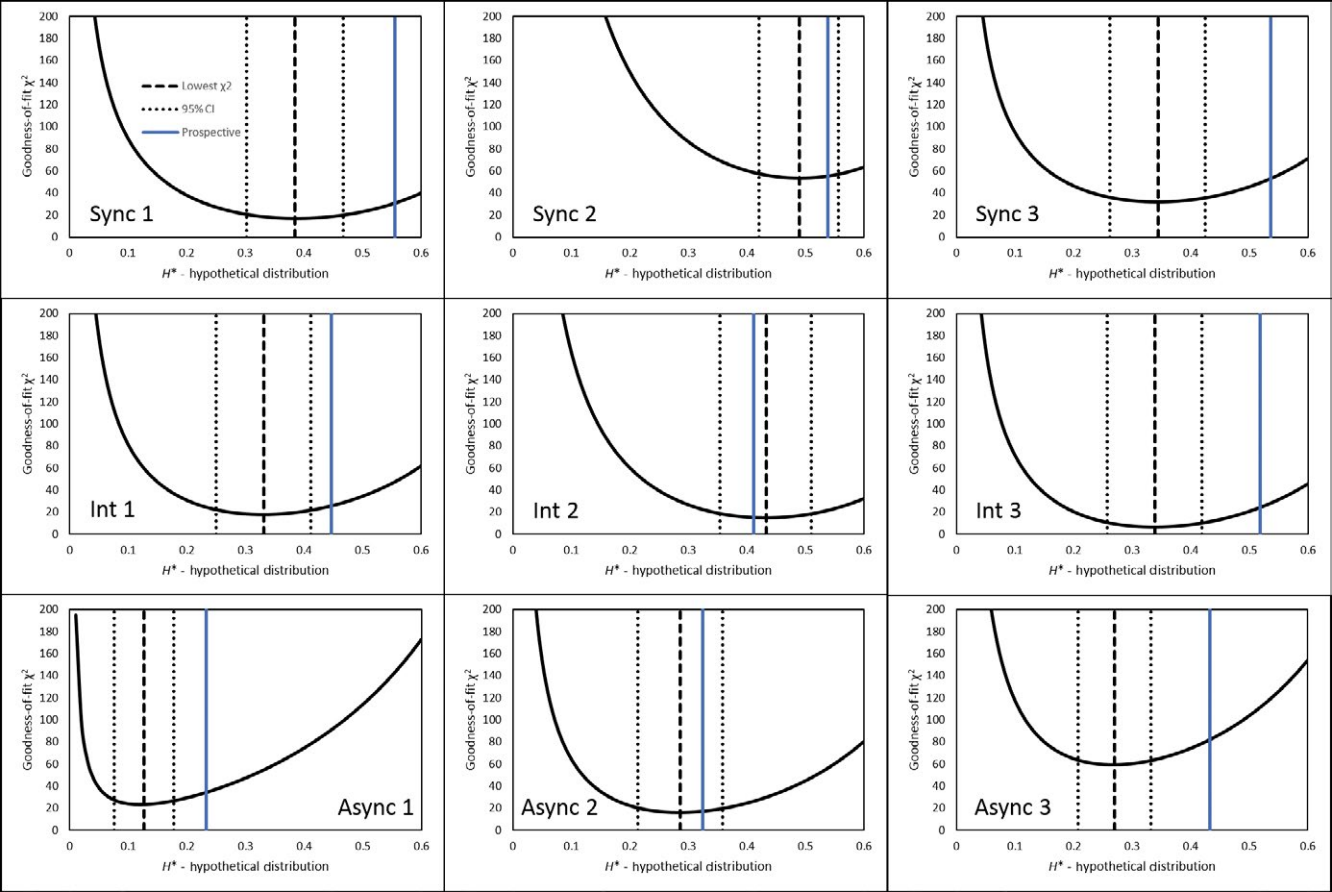
χ^2^ goodness‐of‐fit distributions for residents from each experimental plot with the hybridization value at the minimum χ^2^, confidence intervals, and prospective estimate plotted. Plot number is in the bottom right

**Figure 9:**
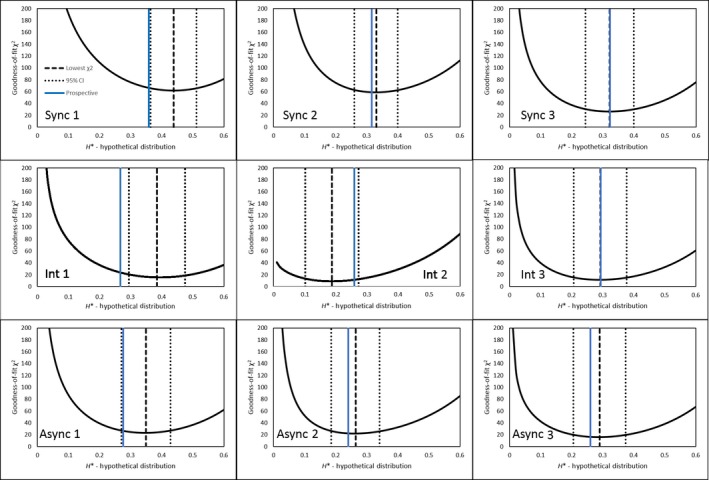
χ^2^ goodness‐of‐fit distributions for migrants from each experimental plot with the hybridization value at the minimum χ^2^, confidence intervals, and prospective estimate plotted. Plot number is in the top left

Retrospective estimates of hybridization by resident plants, obtained by the minimum χ^2^ goodness‐of‐fit method, showed qualitative agreement with prospective estimates (Table 2, Figures 8 & 9)). The purebred‐hybrid synthetic distribution that best fit the flowering time distribution for the seed progeny of residents had mean *H'_R_*values (±95% Confidence interval) of 0.407 ± 0.156, 0.366 ± 0.139, and 0.228 ± 0.127 for the synchronous, intermediate and asynchronous treatments respectively. Thus, realized hybridization in the asynchronous treatment was only ~56% of that under synchrony. The confidence intervals indicate this difference was greater than random, while the intermediate treatment differed from neither of the two more extreme treatments.

**Table 2:** Retrospective estimates of hybridization for each plot across each treatment with treatment means using the minimum χ^2^method

Treatment	Plot	Resident			Migrant		
		*H'_R_*	90% CI	Mean	*H'_M_*	90% CI	Mean
Synchronous	S1	0.388	0.299–0.467		0.439	0.364–0.512	
	S2	0.489	0.420–0.556	0.407 ± 0.156	0.330	0.260–0.400	0.364 ± 0.148
	S3	0.344	0.263–0.424		0.322	0.244–0.399	
Intermediate	I1	0.328	0.288–0.367		0.385	0.294–0.475	
	I2	0.432	0.352–0.511	0.366 ± 0.139	0.189	0.102–0.274	0.288 ± 0.175
	I3	0.339	0.257–0.420		0.292	0.206–0.377	
Asynchronous	A1	0.128	0.075–0.180		0.349	0.270–0.428	
	A2	0.286	0.212–0.359	0.228 ± 0.127	0.264	0.186–0.341	0.301 ± 0.161
	A3	0.270	0.207–0.332		0.290	0.205–0.374	
Resident Only	R1	0.030	0.000–0.083		‐	‐	
	R2	0.000	0.000–0.025	0.042 ± 0.075	‐	‐	‐
	R3	0.096	0.047–0.144		‐	‐	
Migrant Only	M1	‐	‐		0.103	0.056–0.149	
	M2	‐	‐	‐	0.108	0.052–0.163	0.080 ± 0.097
	M3	‐	‐		0.030	0.000–0.084	

Realized hybridization was lower for migrants. However, no change in hybridization with phenological mismatch was detected, based on the broad overlap of confidence intervals among the three treatments (*H'_M_* = 0.364 ± 0.148, 0.288 ± 0.171, and 0.301 ± 0.161 for synchronous, intermediate and asynchronous, respectively (Table 2)).

We note that when the minimum χ^2^ goodness‐of‐fit method was applied to the control plots, neither *H'_R_*nor *H'_M_* were different from the true value of zero (Table 2).

**Figure 10:**
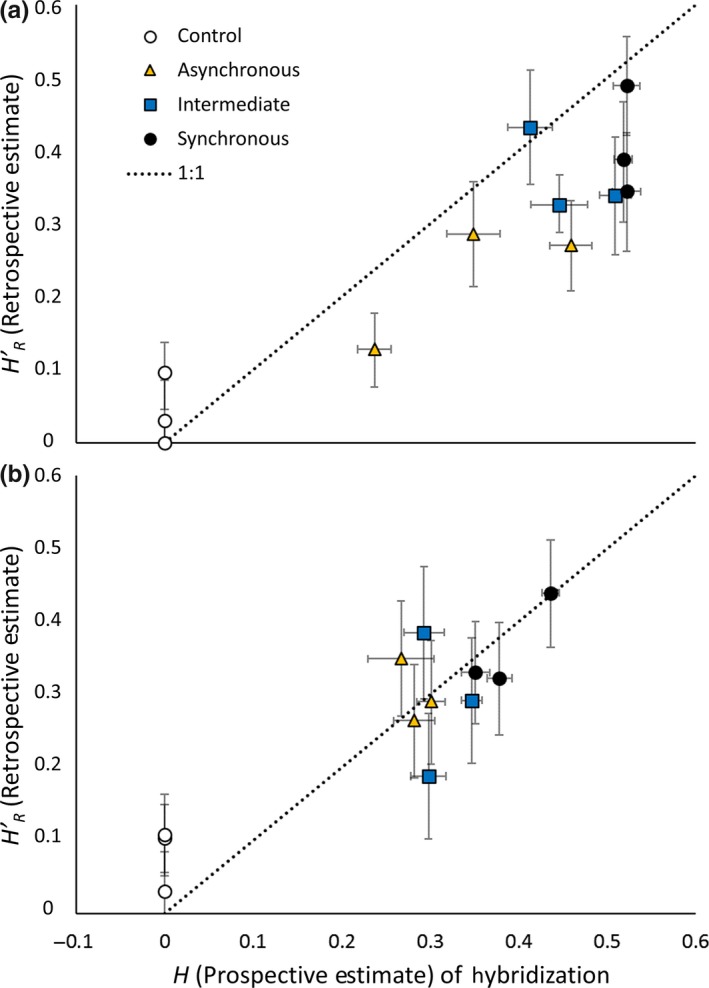
Comparison of Prospective and Retrospective (minimum χ^2^‐goodness‐of‐fit) estimates of hybridization for each plot, for both residents (a) and migrants (b)

Overall, the prospective estimate of hybridization was a reasonable, but biased predictor of the retrospective estimate (Figure 10). The Spearman's rank correlation between the two was 0.84 and 0.68 (one‐tailed *p* < 0.0005 and *p* < 0.01) for residents and migrants respectively, when the control plots are included. If only experimental plots are considered, the rank correlations are 0.64 and 0.27 (one‐tailed *p < *0.05 and *p < *0.24) for resident and migrant plants, respectively. However, *H_R_ *> *H'_R_*in 8 of the 9 experimental plots, and *H_M_ *> *H'_M_* in 6 of the 9.

We evaluated age‐dependent fruit set as a source of bias. Although the flowers that plants produce late in life are less likely to set fruit, the effect was trivial.

The Linear Discriminant Analysis gave the same qualitative results for retrospective estimates of *H'_R_*and *H'_M_* as the minimum χ^2^ goodness‐of‐fit method (Table 3) and correlated with the prospective estimates. These appear biased in the complementary way, overestimating hybridization for migrant plants, but not for residents. However, the LDA method estimated hybridization rates in the control plots to be ~0.2, far greater than the true value of zero. Therefore, this second estimation method appears to be fundamentally biased.

**Table 3:** Retrospective estimates of hybridization for each plot across each treatment with treatment means obtained from the linear discriminant analysis

Treatment	Plot	Resident			Migrant		
		*H'_R_*	90%CI	Mean	*H'_M_*	90%CI	Mean
Synchronous	S1	0.418	0.360–0.475		0.484	0.436–0.554	
	S2	0.572	0.494–0.643	0.487 ± 0.136	0.365	0.309–0.421	0.414 ± 0.116
	S3	0.47	0.399–0.541		0.392	0.333–0.451	
Intermediate	I1	0.424	0.358–0.490		0.466	0.407–0.525	
	I2	0.507	0.441–0.574	0.452 ± 0.131	0.283	0.228–0.342	0.378 ± 0.115
	I3	0.425	0.362–0.490		0.385	0.328–0.440	
Asynchronous	A1	0.195	0.146–0.243		0.413	0.354–0.475	
	A2	0.368	0.306–0.433	0.308 ± 0.121	0.301	0.248–0.357	0.358 ± 0.115
	A3	0.362	0.296–0.431		0.36	0.303–0.418	
Resident only	R1	0.181	0.132–0.237		‐	‐	
	R2	0.103	0.064–0.147	0.182 ± 0.103	‐	‐	‐
	R3	0.262	0.207–0.326		‐	‐	
Migrant only	M1	‐	‐		0.159	0.115–0.200	
	M2	‐	‐	‐	0.179	0.133–0.225	0.172 ± 0.088
	M3	‐	‐		0.179	0.133–0.221	

## 4 DISCUSSION

Gene flow between populations depends on reproductive synchrony, with implications for crop management, conservation, and basic evolutionary processes. In plants, the opportunities for pollen exchange between resident and migrant populations decline with divergence in the flowering schedule. The quantity and quality of pollinator service can affect how many of the remaining opportunities are realized, and thereby cause actual rates of resident–migrant hybridization to differ from those expected from flowering schedules alone. Under controlled hand‐pollination in our greenhouse experiment, predicted and realized hybridization rates strongly agreed. In the field experiment, where plants were exposed to natural pollinators only, realized hybridization rates were correlated to those predicted from flowering schedules, but were lower.

Beyond divergence in the onset of flowerings, schedules for residents and migrants also differed in size and shape. These differences caused asymmetry in hybridization. In the greenhouse, the early‐blooming residents produced more flowers across their schedule than the later migrants, which increased opportunities for the former to transfer pollen to the latter. In the field, the later‐blooming residents flowered over more days than residents, which increased opportunities for migrant‐migrant pollen exchange.

Together, these results suggest that while the opportunity for mating can be a useful indicator of potential gene flow, it may underestimate actual rates of pollen exchange in some environments. Below we explore reasons for quantitative differences between prospective and retrospective hybridization estimates, and more broadly, discuss implications of phenological mismatch.

### 4.1 Lost mating opportunities

The quantitative discrepancy between prospective and retrospective estimates for residents (*H_R_* vs. *H'_R_*) may reflect the loss of mating opportunities under natural pollination. As noted above, non‐random pollen exchange could arise though pollinator preferences for large inflorescences, decline in the per‐flower visitation rate with display size, or foraging responses to flower density. These could cause the proportion of migrant pollen delivered to receptive stigmas to differ from its proportion in the pollen pool on a given day (*q_d_*, Equation ((1)) and ((2)). Day to day changes in weather could have caused pollination intensity to fluctuate, both by directly effecting pollinator foraging, and indirectly by altering floral rewards (Pleasants, 1983; Vicens & Bosch, 2000). Inclement weather during the narrow window of overlap in resident and migrant flowering schedules could thus reduce hybridization. Conversely, unfavorable conditions before and after the overlap window could decrease purebred mating frequency.

Herbivory in the field experiment was very low, but in other cases it could affect mating opportunities, especially if the strength of herbivory varies over time. Many studies have found that herbivory often peaks around peak‐flowering (Elzinga et al., 2007). This could reduce hybridization opportunities when plants flower in synchrony, or increase it when they flower asynchronously, by reducing seed‐set in a biased way. Disease could have similar on‐peak effects, with transmission rates of floral diseases being higher when more flowers are open, or diseases in general which are carried by pollinators (Elzinga et al., 2007). The anther smut *Microbotryum violaceum*, attacks more often earlier in the season, countering the reproductive advantage early‐flowerers have in male function (Biere & Antonovics, 1996). Finally, pollinators may bypass inflorescences with several damaged flowers, leaving the neighboring intact flowers unmated (Krupnick, Weis, & Campbell, 1999).

We note that hybridization rates were asymmetrical in the field experiment; that is, migrant plants produced about as many hybrid offspring as predicted by flowering schedules, while resident plants produced fewer. This asymmetry may have emerged from the selection regime that created the two phenological lines. The linear discriminant analysis showed that plants that flower early have larger flowers (see Appendix S1B). In *Brassica rapa* flower size has been correlated with pollen size (Sarkissian & Harder, 2001), which in turn is correlated with increased siring ability (McCallum & Chang, 2016; Sarkissian & Harder, 2001) and with increased competitiveness of sired seeds within the ovary (Cruzan, 1990). Earlier experiments with the base population for the selection lines also suggested a siring advantage for early flowering (Austen & Weis, 2016). Asymmetric hybridization could alter the evolutionary trajectory if cytoplasmic genes, which are maternally inherited, are under local selection (e.g., Galloway 2005). Additionally, migrants were expected to produce more hybrid offspring in the greenhouse, whereas the opposite was true in the field, owing to them producing more late‐season flowers. This asymmetry in hybrid siring rate could vary across years and locales due to changes in growing season length.

Reduced initial hybridization, as we detected, would not necessarily prevent large‐scale introgression of migrant genes. If the purebred progeny of migrants have sufficient absolute fitness (net reproductive rate), adaptive loci can continue to introgress into the resident genetic background through both migrant × resident and migrant × hybrid matings at a rate dependent on their phenological mismatch. However, local selection may act against migrants, and introgression may be slowed considerably if it acts against migrant phenology itself. The residents that mate with migrants will be those with the most migrant‐like phenology (Weis, 2015). If selection acts against migrant phenology, it will be acting against migrant‐like residents as well, meaning that hybrids will tend to inherit maladaptive phenology alleles from both parents. This “narrows the bridge” over which adaptive migrant alleles pass into the resident population. Any adaptive alleles the migrant may have will likely be in linkage disequilibrium with their maladaptive phenology loci in early generations, further slowing introgression (Soularue & Kremer, 2014).

### 4.2 Implications

The field experiment showed initial hybridization rates can decline with increasing phenological divergence between residents and migrants. Being able to predict potential gene flow between populations based on phenology has implications particularly for agriculture and conservation, but also for invasion biology. Understanding what allows and inhibits hybridization can do much to elucidate how new species arise and diverge. Introgression can also break‐down barriers between species, resulting in the birth of a new species from the collapse of previous ones. Many such species complexes are breaking down on observable time‐scales. The three‐spined stickleback in some lakes has collapsed from two biological species into one due to hybridization (Taylor et al., 2006). On the other hand, weedy sunflowers in highly‐infested fields have evolved to be more divergent phenologically from their croppy cousins (Roumet, Noilhan, Latreille, David, & Muller, 2013). Flowering phenology can pose an effective barrier to hybridization, allowing early‐ and late‐flowering varieties to exist in sympatry with minimal gene flow. (Soliva & Widmer 1999). These divergences in reproductive timing could eventually lead to the development of new species. Crop‐weed gene flow is an important problem in modern agriculture. The transfer of genes into weedy populations is well documented (Roumet et al. 2013, Chèvre et al. 2000, Langevin, Clay, & Grace, 1990, Ellstrand et al., 1999). In particular, gene flow can lead to the spread of herbicide resistance to weedy populations, reducing the effectiveness of herbicides in weed control (Kreiner, Stinchcombe, & Wright, 2017). Multiple herbicide resistance alleles have spread into escaped populations of canola, even outside of crop fields (Knispel, McLachlan, Van Acker, & Friesen, 2008). Of the world's 13 most important food crops, 12 have been found to hybridize with wild populations somewhere in their range (Ellestrand et al., 1999). If selection for crop traits is advantageous, it can lead to genetic swamping in wild populations (Ellestrand et al., 1999; Haygood, Ives, & Andow, 2003). Transgene escape in sunflowers has been shown to lead to increased fecundity and decreased herbivory in wild populations (Snow et al. 2003). Demographic swamping may also occur in instance where large populations of crops successfully breed with wild populations, flooding the ecosystem with unfit hybrids (Haygood et al., 2003). Crops planted in large monocultures are likely to spread their genes to receptive wild populations. Choosing cultivars that flower asynchronously from their wild relatives (Jenczewski, Ronfort, & Chèvre, 2003) can be employed to slow crop‐weed gene flow through phenological divergence (Roumet et al. 2012).

Gene flow is an important factor in the survival of colonizing species. Gene flow to a founder population will prevent founder effects and improve genetic diversity. Understanding how species colonizing novel ecosystems become invasive is critical to preserving global biodiversity (Vitousek, D'antonio, Loope, Rejmanek, & Westbrooks, 1997). Many invasive species do not become invasive immediately after colonization, suggesting that many species are not simply pre‐adapted to invasion (Ewel et al. 1999). Hybridization between native species and an introduced species may cause a native species to become invasive through the input of both additional heterozygosity and novel adaptive variation (Ellstrand & Schierenbeck 2000). Multiple introductions of a species may augment the amount of genetic diversity an invading species has, provided these introductions readily interbreed (Dlugosch & Parker, 2008).

Rising global temperatures may send some narrowly‐adapted populations into decline. A new, unfavorable climate can reduce mean fitness of a local population, reducing net reproductive rate below replacement level, eventually leading to local extinction. If rapid enough, adaptation can restore mean fitness. This process, called “evolutionary rescue”, depends critically on standing genetic variation in the traits under selection (Bell & Gonzalez, 2009; Gomulkiewicz & Shaw, 2013). Evolutionary rescue is at the core of Assisted Gene Flow (AGF), which depends on interbreeding between populations. Hybridization between species and populations can lead to increased diversity and introduce novel traits, and potentially promote evolutionary rescue (Aitken & Whitlock, 2013, Janes & Hamilton, 2017). Gene flow can be inhibited by factors other than phenological divergence, however many of these factors are unlikely in most scenarios where AGF is relevant (Aitken & Whitlock, 2013). Phenological divergence poses a much more significant problem and is rooted in the biology of the conservation targets.

Migrant phenology in its habitat may not reliably indicate phenology after translocation, however. Flowering in many species of plants occurs after accumulating a number of degree‐days above a basal temperature (Forrest & Thomson, 2011). Other plant species have a vernalization requirement, that is, the heat accumulation mechanism is activated in spring after accumulating a particular number of winter chilling degree‐days (e.g., Mimura & Aitken, 2010; Stinchcombe et al., 2004). Fruiting trees, such as almonds, have different chilling requirements, causing them to be very phenologically divergent (Egea, Ortega, & Martínez‐Gómez, P., & Dicenta, F., 2003). In many species, heat accumulation mechanisms are activated only after an environmental cue exceeds the threshold value (Andres & Coupland, 2012), such as when flowering is triggered by photoperiod (e.g., Friedman & Willis, 2013). Long‐day, spring‐flowering species may flower too early when translocated north, while short‐day, summer‐flowering plants may flower too late.

Our experiment demonstrates that reproductive timing has a large, yet predictable, effect on hybridization. While field results are likely to differ from estimates of mating opportunities, our prospective estimate will still give insight into potential for populations to hybridize.

## AUTHOR CONTRIBUTIONS

Colin Bonner and Arthur E. Weis conceived of the presented idea and wrote the manuscript. Sally Erin Westover bred the phenological lines of *Brassica rapa*. Michelle Ho and Nina A. Sokolov contributed to data collection and analysis and assisted in editing the manuscript.

## Data Availability

Dryad repository DOI accession number: https://doi.org/10.5061/dryad.3n6qm3p.
