# Supplementary material for: Estimating the impact of divergent mating phenology between residents and migrants on the potential for gene flow
Source: Ecol Evol. 2019 Mar 12;9(7):3770–83. doi: 10.1002/ece3.5001 (PMC6468075; doi:10.1002/ece3.5001)
Supplement: Supplementary file 1 [file ECE3-9-3770-s001.docx]

**Appendix**

1. **Minimum χ^2^-goodness-of-fit procedure:**

In order to retrospectively estimate hybridization rates in the offspring generation we needed to estimate the phenotypic distribution of flowering time for various hybridization rates. We grew known F_1_ hybrids, purebred residents, and purebred migrants, in the same environment as our experimental plants of unknown parentage. We then binned the frequency of flowering times of each genotype into two-day intervals. Experimental plots were similarly binned for comparison. For example, the distribution for plot 1 residents:

| *Bin* | *Frequency* |
| --- | --- |
| 163 | 13 |
| 165 | 40 |
| 167 | 50 |
| 169 | 51 |
| 171 | 38 |
| 173 | 17 |
| 175 | 12 |
| 177 | 5 |

The three populations of known genotype gave us the phenotypic distribution for theoretical populations composed of 0% and 100% hybrid individuals. In order to determine the hybridization rates of our experimental offspring populations, however, we need to know the phenotypic distribution across the full range of possible hybridization values. We thus created “synthetic distributions” by combining the flowering time frequencies of hybrids and purebreds in varying complementary proportions (i.e. 15% hybrid and 85% purebred). To make sample size equal between the synthetic distributions and each experimental distribution, synthetics were created using densities and then converted to frequencies by multiplying by the number of
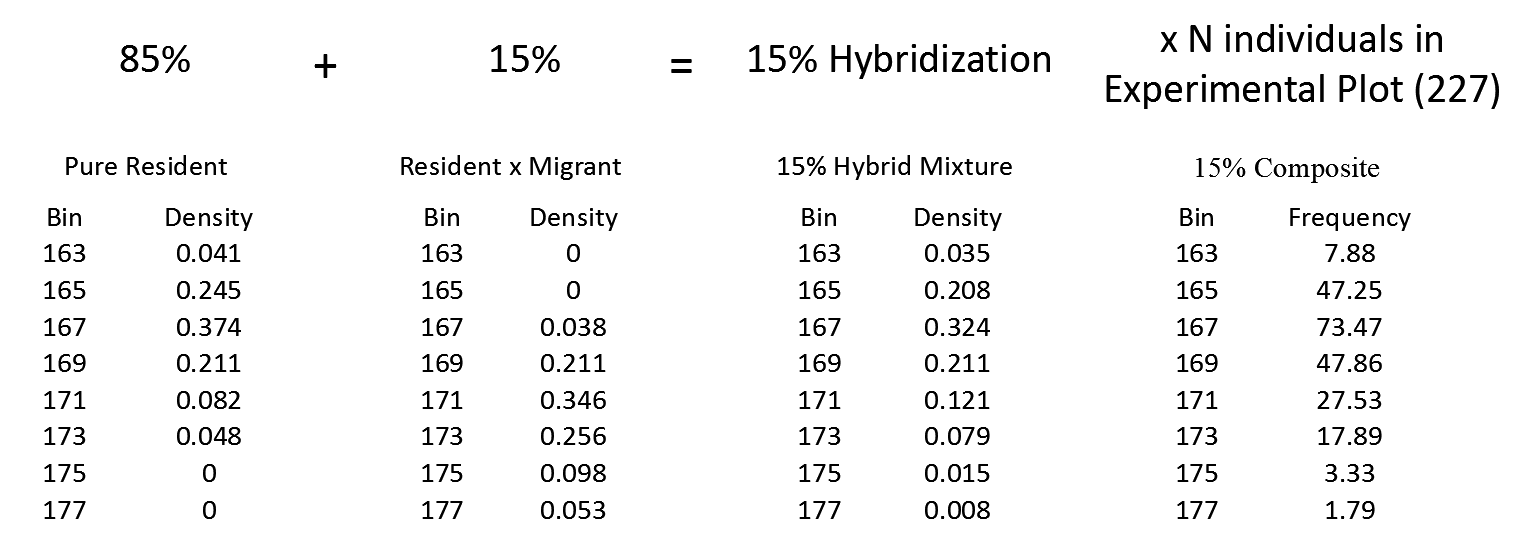
individuals in the experimental plot. For example:

We constructed synthetic distributions for every 1% hybridization between 0% and 50%, and performed a χ^2^-goodness-of-fit test between every synthetic distribution and each observed distribution. When testing against the 0% hybrid distribution, which lacked any individuals flowering later than day 173 in residents, and earlier than day 167 in migrants, any individuals from these days in the observed distribution were pooled together into the 173 and 167 bins respectively. The distribution of χ^2^ values for each synthetic distribution was plotted against the corresponding hybridization rate, and a quadratic regression was then fit to the distribution of χ^2^ values around the minimum in order to interpolate the proportion hybrid to 3 decimal places. Every regression had an R^2^ of at least 0.9, with all but two populations having an R^2^ less than 0.95, and every regression spanned points at least 3.84 unites above the minimum. For example:

In this population, we included hybridization values between 0.28 and 0.37. The regression takes the form y=ax^2^+bx+c, and minimum χ^2^ was interpolated by calculating $\frac{-b}{2a}$. The 95% confidence interval on either side of the minimum was calculated by interpolating the hybridization value at 3.84 χ^2^ above the minimum using the quadratic regression.

1. **Linear Discriminant Analysis:**

Offspring from resident and migrant mothers were analyzed separately for every plot using the linear discriminant analysis. First, a linear discriminant analysis (LDA) was performed on both purebred and hybrid offspring from known crosses as a “training set”. Six traits were measured and used in the LDA: flowering date, stem diameter at the crown, total node number, node number at the crown, stem height (up to the first inflorescence), and corolla width. The LDA predicted the genotype of resident and migrant offspring with 80.1% and 83% accuracy, respectively. The coefficients of each trait are as follows:

| **Resident vs. Hybrid** | | | **Migrant vs. Hybrid** | | |
| --- | --- | --- | --- | --- | --- |
| Resident Coefficients of linear discriminants: | | | Migrant Coefficients of linear discriminants: | | |
|  |  |  |  |  | |
| Flowering date | 0.456412415 |  | Flowering date | | 0.31807968 |
| Diameter | 0.165485339 |  | Diameter | | 0.3992147 |
| Crown node number | 0.284195281 |  | Crown node number | | -0.07578559 |
| Total nodes | -0.066356582 |  | Total nodes | | 0.07746957 |
| Height | 0.003492619 |  | Height | | -0.01607546 |
| Corolla width | -1.073035184 |  | Corolla width | | -0.15787002 |

These coefficients from the training set were then used to predict the number of hybrids in each of the experimental plots for both residents and migrants.
